# Morphology controlling method for amorphous silica nanoparticles and jellyfish-like nanowires and their luminescence properties

**DOI:** 10.1038/srep22459

**Published:** 2016-03-04

**Authors:** Haitao Liu, Zhaohui Huang, Juntong Huang, Song Xu, Minghao Fang, Yan-gai Liu, Xiaowen Wu, Shaowei Zhang

**Affiliations:** 1 School of Materials Science and Technology, Beijing Key Laboratory of Materials Utilization of Nonmetallic Minerals and Solid Wastes, National Laboratory of Mineral Materials, China University of Geosciences (Beijing), 100083, P. R. China; 2 College of Engineering, Mathematics and Physical Sciences, University of Exeter, Exeter EX4 4QF, UK; 3 School of Engineering and Technology, China University of Geosciences (Beijing), 100083, P. R. China; 4 Department of Mechanical Engineering, University College London, Torrington Place, London WC1E 7JE, UK

## Abstract

Uniform silica nanoparticles and jellyfish-like nanowires were synthesized by a chemical vapour deposition method on Si substrates treated without and with Ni(NO_3_)_2_, using silicon powder as the source material. Composition and structural characterization using field emission scanning electron microscopy, transmission electron microscopy, energy dispersive X-ray spectroscopy and fourier-transform infrared spectroscopy showed that the as-prepared products were silica nanoparticles and nanowires which have amorphous structures. The form of nanoparticles should be related to gas-phase nucleation procedure. The growth of the nanowires was in accordance with vapour-liquid-solid mechanism, followed by Ostwald ripening to form the jellyfish-like morphology. Photoluminescence and cathodoluminescence measurements showed that the silica products excited by different light sources show different luminescence properties. The emission spectra of both silica nanoparticles and nanowires are due to the neutral oxygen vacancies (≡Si-Si≡). The as-synthesized silica with controlled morphology can find potential applications in future nanodevices with tailorable photoelectric properties.

Nanomaterials, such as nanoparticles (NPs)^1^,^2^,^3^, nanowires (NWs)^4^,^5^,^6^ and nanofilms (NFs)^7^,^8^,^9^, have been received steadily growing attention as a result of their peculiar and fascination properties. They offer great application prospects in various fields, including biomedical, electrical and optical fields, *etc.*^10^,^11^ The morphologies of nanomaterials are of vital importance to their potential applications in different scopes. Efforts should be focused on the search for controlling synthesis of multifarious morphologies of one certain material by low cost and simple routes. In the past few years, researchers have witnessed significant advances in the morphology control synthesis of diverse nanomaterials including NPs, NWs, NFs, nanoporous materials, *etc.*^12^,^13^,^14^,^15^,^16^

As one type of the most important luminescence materials, silica nanomaterials have tremendous promise as indicators and photon sources for a number of biotechnological and information technology applications such as biological imaging, sensor technology, microarrays, and optical computing^17^,^18^,^19^,^20^,^21^. The morphology and size controlled products can be specifically designed to satisfy different specifications for their applications. For instance, many researches focused on the biomedical applications of silica NPs as a result of their biocompatibility, low toxicity and scalable availability^17^,^20^. Silica NWs have potential application in high-resolution optical heads of scanning near-field optical microscopes and low dimensional wave-guides in future integrated optical devices for their intense and stable blue light emission at room temperature^22^,^23^,^24^.

Here we report a simple process which could control synthesis of silica NPs and NWs. Silicon powder and p(111) Si substrate was used as the starting materials. The use of a catalyst is the key factor in the morphology control. The detailed growth mechanism for the jellyfish-like NWs morphology was discussed. Photoluminescence (PL) and Cathodoluminescence (CL) were used to characterize optical properties of the as-prepared NPs and NWs products, which are of benefit to understanding not only the relationship between morphology and luminescence properties of silica nanostructures, but also their optical properties in different excitation source. We think these researches could provide a facile method for the synthesis of silica NPs and NWs, and are a great help for the application of silica nanostructures in fluorescence labeling medicine or new generation nanodevices with tunable photoelectric properties.

## Results and Discussion

**Figure 1:**
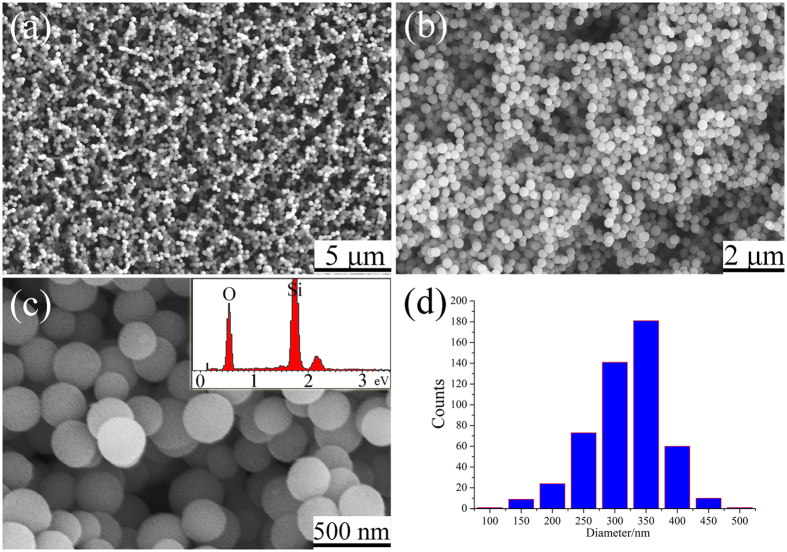
Typical low magnification FESEM image (**a** and **b**) and high magnification FESEM image (**c**) of the as-prepared products. The inset in (**b**) is EDS spectrum of the NPs. (**d**) Size distribution of as-synthesized silica NPs.

In this research, without using templates, relatively uniform silica nanoparticles can be synthesized on Si wafer when the Si substrate was untreated by Ni(NO_3_)_2_ solution. Analysis by FESEM showed that in the absence of template, silica could form on silicon substrate as preferentially loose particles (Fig. 1a–c) with a nanosized diameter concentrated within the range of ~250–400 nm (Fig. 1d). The nanoparticles were composed of 32.1% Si and 67.9% O as revealed by EDS (inset in Fig. 1c), corresponding to the stoichiometric composition of SiO_2_.

**Figure 2:**
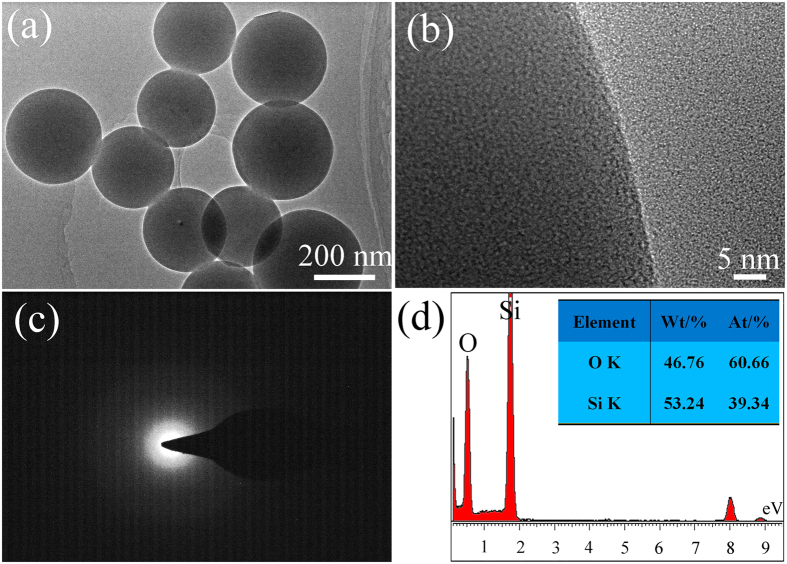
(**a**) TEM image, (**b**) HRTEM image and (**c**) SAED pattern of the nanospheres. (**d**) EDS spectrum taken from a single nanoparticle.

Figure 2a is a typical low magnification TEM image, showing the typical ball-like geometry of silica with diameter range from 200 nm–300 nm. High-resolution TEM analysis (Fig. 2b) of silica particles provided an evidence for their amorphous structure. The corresponding SAED pattern of the ball-like particle is shown in Fig. 2c, which proofed their amorphous structure. The EDS analysis reveals that the NP was composed of 39.34% Si and 60.66% O. Together with HRTEM, SAED and the EDS results measured in SEM and TEM, we roughly considered the as-prepared ball-like products were amorphous silica NPs.

**Figure 3:**
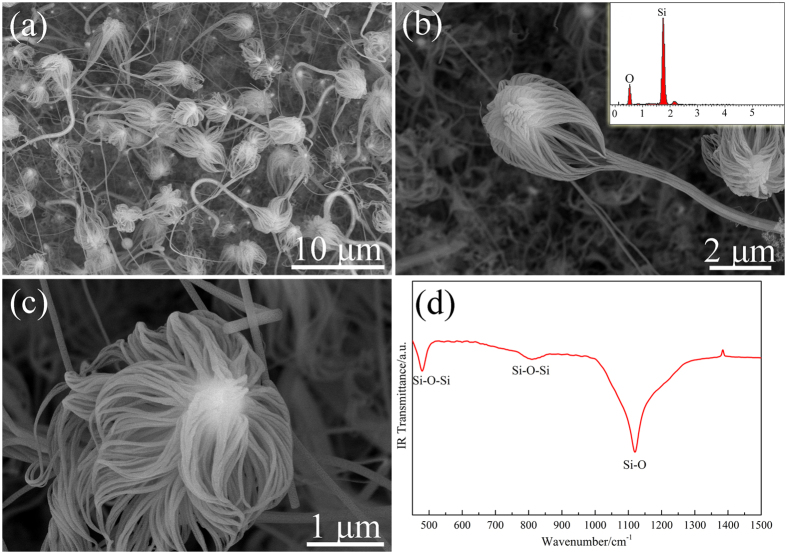
(**a**) Low magnification FESEM image and (**b,c**) high magnification FESEM images of the jellyfish-like NWs product synthesized on a silicon wafer. The inset in (**b**) is EDS spectrum taken from the NWs area in (**b**). (**d**) FT-IR absorption spectrum of the jellyfish-like product.

As Fig. 3a shows, jellyfish-like structured products were prepared when Si wafer was treated by aqueous Ni(NO_3_)_2_ solution before the CVD reaction. High magnification FESEM images (Fig. 3b,c) show that the jellyfish-like structures were composed of numerous NWs. EDS spectrum inset in Fig. 3b reveals the NWs composition mainly contain Si and O elements. Furthermore, FT-IR spectroscopy was also used to confirm the composition of the jellyfish products. As shown in Fig. 3d, the peaks at 479 cm^−1^ and 812 cm^−1^ were correspond to Si-O-Si stretching vibration of amorphous SiO_x_. The peak at around 1119 cm^−1^ was attributed to Si-O stretching vibration. Therefore, the jellyfish-like nanostructures were also silica products.

**Figure 4:**
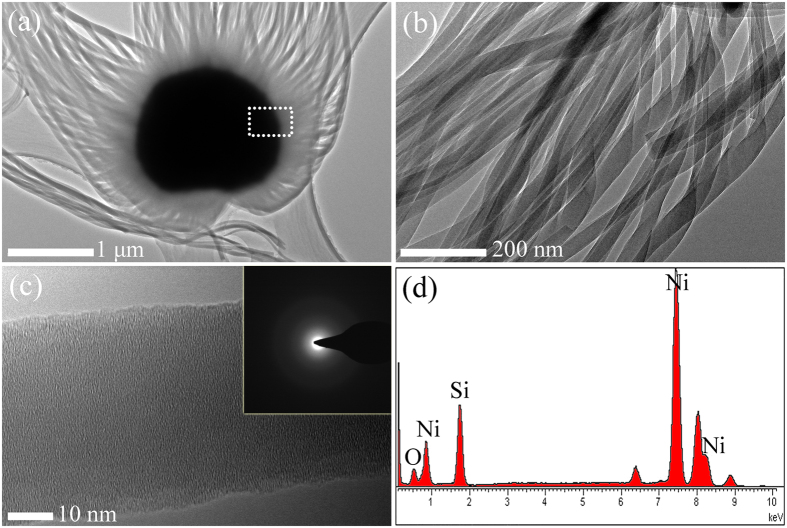
Conventional TEM image of the jellyfish-like NWs, where the metal catalyst can be easily distinguished from the nanowires. (**b**) A typical TEM image of the NWs under low magnification. (**c**) A typical HRTEM image of a single NW and the inset is the corresponding SAED pattern. (**d**) EDS spectrum taken from the marked area in (**a**).

Furthermore, the jellyfish-like products were further characterized using TEM equipped with EDS. Figure 4a shows the typical TEM image of the silica NWs, where the spherical catalyst particle attached to the tip of the “jellyfish head” (NWs) had a diameter ~1.5 μm. The diameter of the NWs as shown in Fig. 4b was 35–50 nm and very uniform. The HRTEM image of the NWs is shown in Fig. 4c, which were similar to the NPs, combined with the SAED image inset in Fig. 4c, revealed the NWs an amorphous phase. The EDS spectrum (Fig. 4d) of the “head of the jellyfish” recorded from the marked area in Fig. 4a depicts the area was composed of Ni, Si, O, which implied that Ni provided a site for the initial nucleation and successive growth of amorphous silica NWs.

In the growth mechanisms of silica NPs and NWs prepared by thermal CVD procedure, the involvement of vapour phase and Ni-catalyst played important roles in their formation process. In this work, before argon was introduced into the experimental setup, the vapour was difficult to expel fully from the system by the rotary pump. When the sample was heated to 1200 °C, the residual oxygen (O_2_ and H_2_O) in the furnace and the oxygen released from the refractory lining can react with the silicon powder following reaction (1)^25^,^26^.


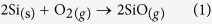


Furthermore, SiO gas can also be formed according to the following reaction (2) on account of the SiO_2_ thin film existed on the surface of Si substrate.


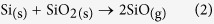


The as-formed SiO gas was transported to the substrate position by the carrier gas. As it is known, SiO is metastable and can be decomposed into SiO_2_ and Si. As we previously mentioned, a certain amount of oxygen remained in the furnace, which reacted with Si vapour to further generate SiO_x_^23^.

In conventional CVD procedure, the vapors were transported and condensed on to a solid substrate surface which was placed in a lower temperature zone^27^. Different from this classical growth mechanism, a few studies have previously been reported in which nanoparticles was formed through gas-phase nucleation procedure. Gas-phase nucleation procedure is also an important process for the synthesis of nanoparticles^28^,^29^,^30^. By using both approaches, the morphology of the silica products in this research can be controlled effectively.

For the generating of silica nanoparticles, a gas-phase nucleation dominated mechanism was proposed. When the as-generated SiO gas diffused to the lower temperature zone, SiO_x_ clusters were generated and fell down on the substrate surface. No 1D silica structures were observed on the substrate without catalyst (as shown in Fig. 1a,b). It indicates that the silica NPs cannot grown anisotropy on the surface of the substrate. Correlative researches indicated that the partial pressure of precursor gaseous has an important role in determining the diameters of the nanoparticles. Nevertheless, the exact growth mechanism of silica NPs still needs to be further investigated.

As for one dimensional (1D) nanostructures, the vapour-liquid-solid (VLS) growth mechanism is most widely employed^27^. The mechanism uses foreign element catalytic agent to medicate the growth^31^. VLS methods are used to prepare various kinds of 1D materials such as Si, Ge, GaN, GaAs, ZnO, ZnS, MgO, SiO_2_, *etc.*^27^

In this research, on account of Ni-catalyst (as revealed in Fig. 4a), VLS procedure dominated the growth of silica NWs. However, a small number of silica NPs were also observed in this process as shown in Figure S1. This phenomenon is results of two mechanisms (gas-phase nucleation and VLS). Due to the addition of Ni-catalyst, the nucleation barrier on the substrate surface was reduced. Ni-Si-O eutectic alloy was formed when SiO gas diffused onto the surface of the substrate. With the diffusion of SiO, when the alloy reached supersaturation condition, the SiO_x_ nuclei were generated and grew anisotropy. In addition, a type of jellyfish-like 1D nanostructure is produced in the present work rather than conventional single NWs. The Ostwald ripening process is considered (the detailed descriptions can be found in Supporting Informations). Figure 3a and b clearly revealed that the primary nanowires grown in the “head” of the “jellyfish” aggregate along the lateral direction to form nanowire bundles. Therefore, by using the aforementioned procedures, the morphology of the silica products can be controlled.

**Figure 5:**
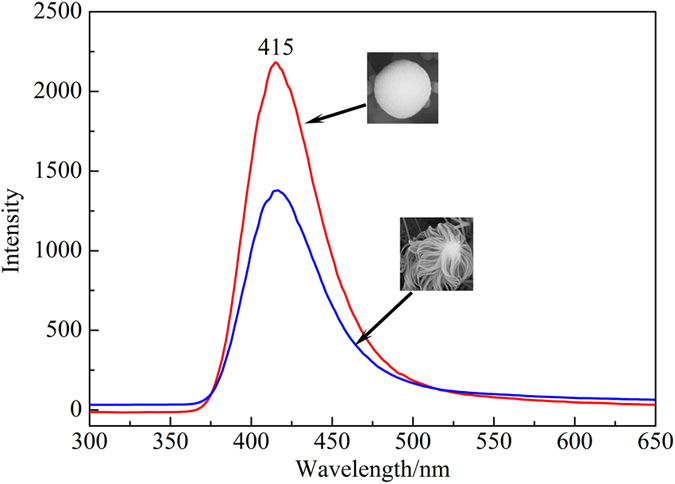
The emission spectra of the as-obtained silica NPs and jellyfish-like NWs.

In order to characterize the difference of optical properties between NPs and NWs, their PL and CL spectra were recorded at room temperature. Figure 5 presents the PL spectra of the as-prepared NPs and NWs using a Xe lamp (254 nm) as the excitation source. Both the NPs and NWs show a blue emission band at 415 nm (2.99 eV). Furthermore, the peak intensity of the NPs is much higher than that of the nanowires. This suggests that the stimulated area of the NPs is much larger than the NWs.

**Figure 6:**
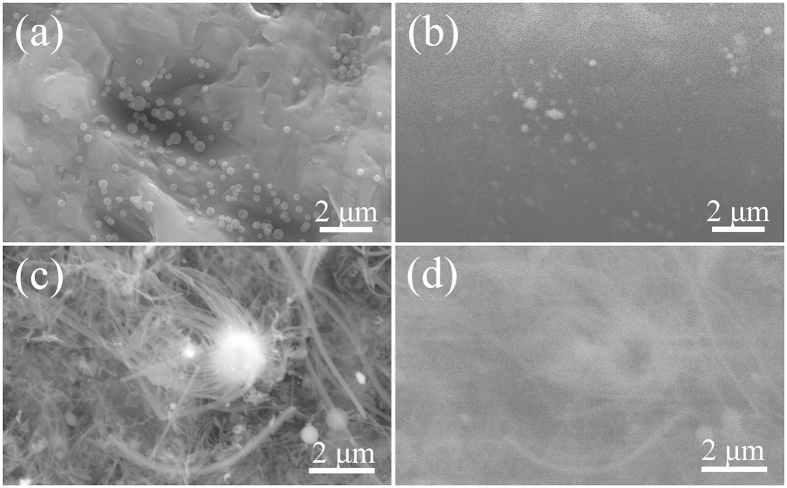
SEM images (**a** and **c**) and their corresponding CL images (**b** and **d**) of the as-obtained silica NPs and NWs. The images were obtained with a focused electron beam at an accelerating voltage of 30 kV.

Figure 6 shows together NPs and NWs SEM images (Fig. 6a,c) and homologous CL images. From the CL images, the contrast from the nanostructures (NPs and NWs) displayed clear bright variations.

**Figure 7:**
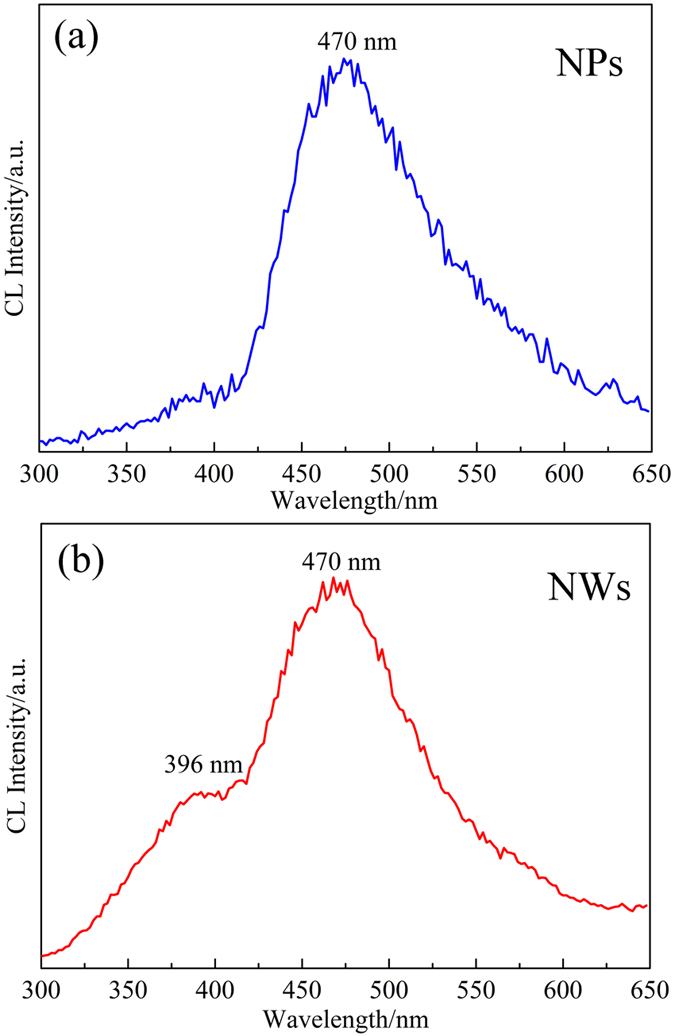
Room temperature CL spectra of the as-synthesized silica nanostructures. (**a**) NPs; (**b**) NWs.

To investigate the luminescence properties of these two types of nanostructures in detail, CL spectra (as shown in Fig. 7) were also collected at an accelerating voltage of 30 kV. However, different emission spectra were obtained compared with their PL spectra. All of the silica nanostructures demonstrated a blue emission centered at ~470 nm (2.64 eV). The NWs’ spectra show an emission shoulder at ~396 nm (3.13 eV). Up to now, a number of researches have been carried out to investigate the luminescence of SiO_x_ NPs^32^ and NWs^33^. Blue/green emissions ranged from 380 nm (3.26 eV) to 530 nm (2.34 eV) wavelengths have been reported for silica nanostructures^23^,^24^,^33^,^34^. As previously reported, the luminescence peaks at around 410 nm (3.02 eV) ~470 nm (2.64 eV) were attributed to the neutral oxygen vacancy (≡Si-Si≡) arising from such oxygen deficiency^35^,^36^,^37^. The peak positions including PL and CL spectra in our silica NPs and NWs are in general agreement with the previously reported values. Hence, both the PL spectra and CL spectra are caused by the emission of the excited oxygen vacancies. In CL measurements, under an accelerating voltage of 30 kV, the electrons could inject into a hundred of nanometers even several micrometers in depth into the samples. This may cause the different emission peaks between these two kinds of tests, even measured the same products. As previous reports demonstrated, silica nanostructures showed a varying degree of oxygen deficiency, but the emission peaks around 410 nm (3.02 eV) ~470 nm (2.64 eV) are all attributed to the neutral oxygen vacancy (≡Si-Si≡)^33^. In this research, under an accelerating voltage of 30 kV, the electrons could inject into a hundred of nanometres even several micrometers in depth into the samples. This may cause a different dominated luminescence mechanism between PL and CL measurements. Based on the above analysis, we roughly considered that the number and depth of the neutral oxygen vacancies which are excited in silica nanostructures may affect the emission peak position. Compared the CL spectra between NPs and NWs, the appearance of the 396 nm shoulder in NWs CL spectrum may due to the intrinsic diamagnetic defect center^38^ or the 1D structure. From the PL and CL results, it can be concluded that the luminescence property of the silica nanostructures can be changed along with the excitation light sources. The changeable emission properties of the silica nanostructures are of significant interest for their potential application in new photoelectric nanodevices. Especially the luminescence properties of the NPs make it is possible to apply in fluorescence labeling medicine field.

## Conclusions

In conclusion, morphology control synthesis of uniform silica nanoparticles (NPs) and jellyfish-like nanowires (NWs) has been achieved using a chemical vapour deposition (CVD) method. Structural characterization indicates that both the NPs and NWs have an amorphous structure. The form of NPs may be related to gas-phase nucleation procedure, and the growth of the nanowires are in accordance with vapour-liquid-solid (VLS) mechanism. And the jellyfish morphology of the as-prepared silica nanowires is related to Ostwald ripening effect. PL and CL measurements indicate that the emission spectra of both silica NPs and NWs are due to the neutral oxygen vacancy (≡Si-Si≡) existed in these nanostructures. Interestingly, the luminescence property of the silica nanostructures can be changed along with the excitation light sources. This research provides a simple method in morphology control synthesis of silica NPs and NWs. The outstanding optical properties make them possess considerable potential application prospect in fluorescence labeling medicine field, photoelectric nanodevices *etc.*

## Methods

The experimental setup consists of a horizontal high-temperature tube furnace, an alumina tube, a rotary pump system, and a gas controlling system. Silica NPs and jellyfish-like NWs were synthesized directly on silicon substrate by a simple CVD procedure, using high-purity argon as carrier gas. A p-type Si (111) substrate of size 2 cm × 2 cm was ultrasonically cleaned by acetone and ethanol for 10 minutes each, then dried in air condition. In a typical synthesis reaction, 1 g silicon powder was placed in one end of an alumina boat, and Si substrates (treated without or with several drops of aqueous 0.1 m Ni(NO_3_)_2_ solution, the solution was dispersed and air dried) were placed on the opposite end. After that, the fixed alumina boat was placed in the center of a tube furnace, it is worth noted that the silicon powder end was placed in the air inlet end. The experimental setup was evacuated to 10 Pa by a rotary pump and argon (purity 99.999% (v/v)) was introduced until the furnace pressure reached 0.14 MPa. Then the setup was heated in a programmed way, from room temperature to 1000 °C at 10 °C/min, from 1000 °C to 1200 °C at 3 °C/min and maintained there for 3 h.

The as-prepared products were characterized by field emission scanning electron microscopy (FESEM, Hitach S4800, Japan) and transmission electron microscopy (TEM/HRTEM, FEI-Tecnai-G^2^-F30, America) equipped with energy dispersive X-ray spectroscopy (EDS). Fourier-transform infrared spectroscopy (FT-IR) data were collected with a Nicolet IR100/200 spectrophotometer over the wavenumber range of 450–1500 cm^−1^. After the phase and structure examinations, PL spectra of the as-prepared nanostructures were collected with a fluorescence spectrophotometer (Hitachi F-4600, Japan) using a Xe lamp excitation. CL images and spectra were determined by using an ultrahigh vacuum scanning electron microscope (UHV-SEM) equipped with a Gemini electron gun (Omicron, Germany) and a CL detector (Gatan mono 3 plus) at an accelerating voltage of 30 kV. All PL and CL images and spectra were collected at room temperature under the same conditions to ensure convictive comparsion.

## Additional Information

**How to cite this article**: Liu, H. *et al.* Morphology controlling method for amorphous silica nanoparticles and jellyfish-like nanowires and their luminescence properties. *Sci. Rep.* **6**, 22459; doi: 10.1038/srep22459 (2016).

## Supplementary Material

Supplementary Information
