# Supplementary material for: Morphology controlling method for amorphous silica nanoparticles and jellyfish-like nanowires and their luminescence properties
Source: Sci Rep. 2016 Mar 4;6:22459. doi: 10.1038/srep22459 (PMC4778036; doi:10.1038/srep22459)
Supplement: Supplementary Information [file srep22459-s1.doc]

**Supporting Information**

Morphology controlling method for amorphous silica nanoparticles and jellyfish-like nanowires and their luminescence properties

*Haitao Liu,^a^ Zhaohui Huang,^*a^ Juntong Huang,^b^ Song, Xu^c^, Minghao Fang,^a^ Yan-gai Liu,^a^ Xiaowen Wu,^a,d^ Shaowei Zhang^b^*

^a^ School of Materials Science and Technology, Beijing Key Laboratory of Materials Utilization of Nonmetallic Minerals and Solid Wastes, National Laboratory of Mineral Materials, China University of Geosciences (Beijing), 100083, P. R. China.

^b^College of Engineering, Mathematics and Physical Sciences, University of Exeter, Exeter EX4 4QF, UK.

^c^ School of Engineering and Technology, China University of Geosciences (Beijing), 100083, P. R. China.

^d^Department of Mechanical Engineering, University College London, Torrington Place, London WC1E 7JE, UK.

*huang118@cugb.edu.cn


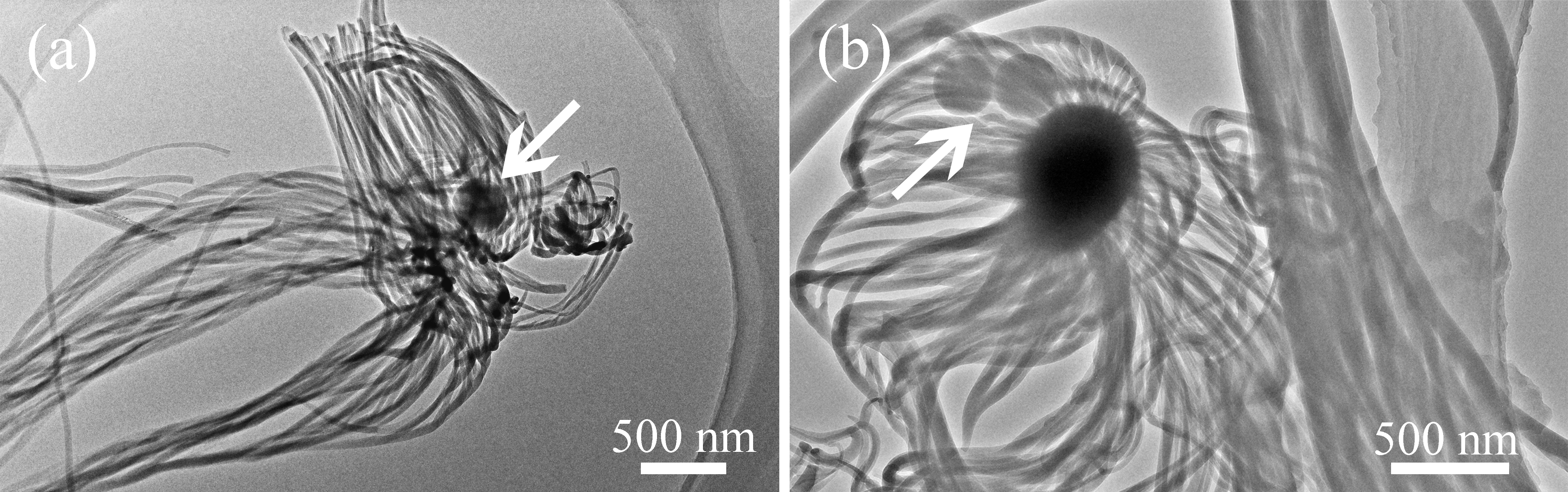


Figure S1 (a-b) Typical TEM images of the jelly-fish NWs, where the nanospheres can be easily distinguished from the nanowires.

**Growth mechanism of jellyfish-like nanowires**

In this research, on account of Ni-catalyst (as revealed in Figure 4a), VLS procedure dominated the growth of silica NWs. As shown in Figure S2, with the growing of nanowires, the primary nanowires aggregate along the lateral direction to form nanowire bundles. This phenomenon can be regarded as one kind of Ostwald ripening process.

Ostwald ripening process is an observed phenomenon that describes the change of an inhomogeneous structure over time. Thermodynamically, this process occurs because larger particles are more energetically favored than smaller particles.^1^ This stems from the fact that molecules on the surface of a particle are energetically less stable than the ones in the interior especially in nanoscale.

**
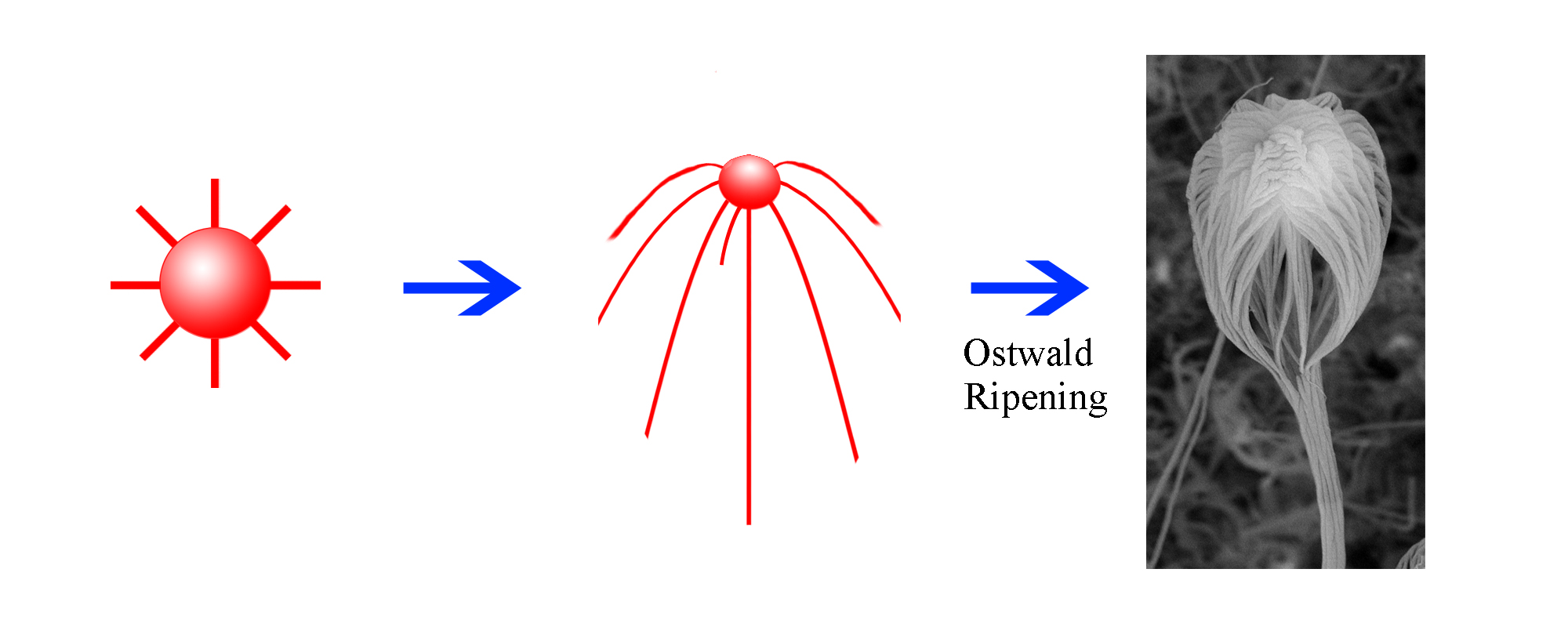
**

Figure S2 Schematic illustration for the growth of jellyfish-like nanowires

**References**

1. Ratke L., Voorhees P W. Growth and coarsening: Ostwald ripening in material processing. *Springer Science & Business Media*, pp. 117-118 (2013).
